# Editorial: Adrenocortical carcinoma: advancing treatment beyond surgery for a rare disease

**DOI:** 10.3389/fendo.2025.1589785

**Published:** 2025-04-24

**Authors:** Julie Hallanger Johnson

**Affiliations:** Division of Endocrinology, Diabetes, Metabolism, and Nutrition, Mayo Clinic, Rochester, MN, United States

**Keywords:** adrenal cancer, Wnt/β -catenin, mitotane, radiation therapy, BET (bromodomain and extra-terminal) inhibitor

In November 2024, an international group of scientists, physicians, and patients gathered in a conference room at the M. D. Anderson Cancer Center (Houston, TX, USA) around one hope—better treatment options for the devastating disease of adrenocortical carcinoma. This was the 9^th^ International Adrenal Cancer Symposium. The group was focused on this disease and its treatments and trying to better understand it to offer improved treatments (1). To date, treatments beyond surgery for this disease have been limited to challenging options with both poor tolerability for patients and their caregivers and with low response rates. Therefore, this Research Topic is of great importance to the community of patients, scientists, and physicians working to improve outcomes for this aggressive cancer.

Adrenal cancer itself is considered rare since it is reported to have an incidence of approximately 1 person per million. Many patients are diagnosed late with Stage III and IV malignancy without an immediate option for surgical cure due to the sinister nature of this particular cancer. This has led to the search for better systemic therapies, as the response rate to the current standard of care therapy with mitotane plus etoposide, doxurubicin, and cisplatin was approximately 23% in the FIRM-ACT trial (2). Unfortunately, progress has been slow in part due to the rarity of the disease. We believe that any opportunity to advance science and clinical care is critical. Therefore, this collection of articles is meant to help advance the current state and increase knowledge to improve outcomes for patients with advanced adrenocortical carcinoma.

These articles range from research on specific mutations to prognostic predictors in a single-center review, to the neurological tolerance of mitotane and even the impact of radiation therapy. Beginning with a better understanding of the pathophysiology of the disease, Tai and Shang provided an elegant discussion regarding Wnt/β -catenin signaling and potential therapeutic targets in ACC. The understanding of this pathway in ACC will allow further exploration of these potential targets in the 30% of patients with ACC with Wnt/β -catenin alterations (3, 4).

Lalli provided additional insight into the mechanism of NR5A1 and Wnt signaling in ACC, adding to the discussion on therapeutics.

Situ et al. described the role of the bromodomain and extracellular terminal (BET) family in ACC. The idea is that if this role is confirmed, some inhibitors could be used as targeted therapy. They found that BRD4 expression is upregulated in ACC and that its expression correlates with disease staging. In addition, they explored other genes whose expression levels correlated with the expression of BRD2, BRD3, and BRD4, deepening our understanding of this disease.

Several articles have been included with clinical treatment highlights—from mitotane toxicities and radiation impact on outcomes to case report of immunotherapy success and a review of single-center experience treating ACC in Taiwan. Mormando et al. described measurements using neurological tools such as EEG to measure neurotoxicity of elevated mitotane levels. This highlights one of the challenges of navigating ACC treatment with mitotane and the ongoing importance of monitoring levels and managing toxicities.

Wu et al. describe their single-center experience with adjuvant radiation therapy in a retrospective analysis of 105 patients, 46 of whom received adjuvant radiation therapy. Interestingly, in patients with early-stage disease (ENSAT I/II), adjuvant radiation therapy improved 3-year overall and disease-free survival. However, in further subgroup analyses, patients matched for Ki67 and stage did not suggest that radiation had an impact on survival. The authors proposed additional prospective studies to address this and other studies’ varied findings in the use of adjuvant radiation therapy for ACC.

Pan et al. described their single-center experience with 67 patients with adrenocortical carcinoma. They confirmed that ENSAT staging correlates with overall survival. In addition, large vessel invasion was a predictor of poorer overall survival. Mitotane use improved outcomes in patients with Stage IV disease.

Charles et al. reported a successful case of neoadjuvant combination of the cytotoxic T- lymphocyte-associated protein 4 (CTLA-4) inhibitor ipilimumab with the programmed-death 1 (PD-1) inhibitor therapy nivolumab. This was a case in which the initial diagnosis was incorrect, but it led to a successful resection with no evidence of disease one year later. The authors raised the question of whether combination immunotherapy might be more promising in the future than previous studies have suggested.

The above offer a glimmer of hope for this rare disease.
